# New Directions in 3D Medical Modeling: 3D-Printing Anatomy and Functions in Neurosurgical Planning

**DOI:** 10.1155/2017/1439643

**Published:** 2017-06-08

**Authors:** Paolo Gargiulo, Íris Árnadóttir, Magnús Gíslason, Kyle Edmunds, Ingvar Ólafsson

**Affiliations:** ^1^Institute of Biomedical and Neural Engineering/Medical Technology Center, Reykjavik University and Landspitali University Hospital, Menntavegi 1, 101 Reykjavik, Iceland; ^2^Department of Neurosurgery, Landspitali University Hospital, Áland, 108 Reykjavik, Iceland

## Abstract

This paper illustrates the feasibility and utility of combining cranial anatomy and brain function on the same 3D-printed model, as evidenced by a neurosurgical planning case study of a 29-year-old female patient with a low-grade frontal-lobe glioma. We herein report the rapid prototyping methodology utilized in conjunction with surgical navigation to prepare and plan a complex neurosurgery. The method introduced here combines CT and MRI images with DTI tractography, while using various image segmentation protocols to 3D model the skull base, tumor, and five eloquent fiber tracts. This 3D model is rapid-prototyped and coregistered with patient images and a reported surgical navigation system, establishing a clear link between the printed model and surgical navigation. This methodology highlights the potential for advanced neurosurgical preparation, which can begin before the patient enters the operation theatre. Moreover, the work presented here demonstrates the workflow developed at the National University Hospital of Iceland, Landspitali, focusing on the processes of anatomy segmentation, fiber tract extrapolation, MRI/CT registration, and 3D printing. Furthermore, we present a qualitative and quantitative assessment for fiber tract generation in a case study where these processes are applied in the preparation of brain tumor resection surgery.

## 1. Introduction

Three-dimensional (3D) modeling and rapid prototyping technologies have recently shown great utility in a wide variety of applications in medicine and surgery [1, 2]. In principle, the 3D recapitulation of patient-specific anatomical features provides surgeons with an immediate and intuitive understanding of even the most complex anatomical morphologies, enabling accurate planning and emulation of a host of surgical procedures [3, 4]. Indeed, the employment of these 3D anatomical models is additionally being considered for a host of implantation procedures, such as dental crowning, craniofacial reconstruction, and tissue regeneration via biological scaffolds [5–7].

Kodama et al. reported the birth of 3D rapid prototyping in 1981 [8], and the first use of the technology in support of surgical planning was reported by Anderl et al. in 1994 [9]. Since then, improvements in medical imaging modalities, such as CT and MRI, have driven both the clinical interest and academic development of 3D rapid prototyping in a medical context. Modern rapid prototyping enables the construction of anatomical models with layer thicknesses on the order of microns, and with concurrent advancements in medical image contrast segmentation, these models are able to recapitulate external and internal anatomical morphologies to high degrees of precision. The utilization of rapid prototyping models incurs a host of benefits to many surgical fields, which include improving surgical planning, enhancing diagnostic quality, decreasing patient exposure time to general anesthesia, decreasing patient blood loss, and shortening wound exposure time [10].

**Figure 1:**
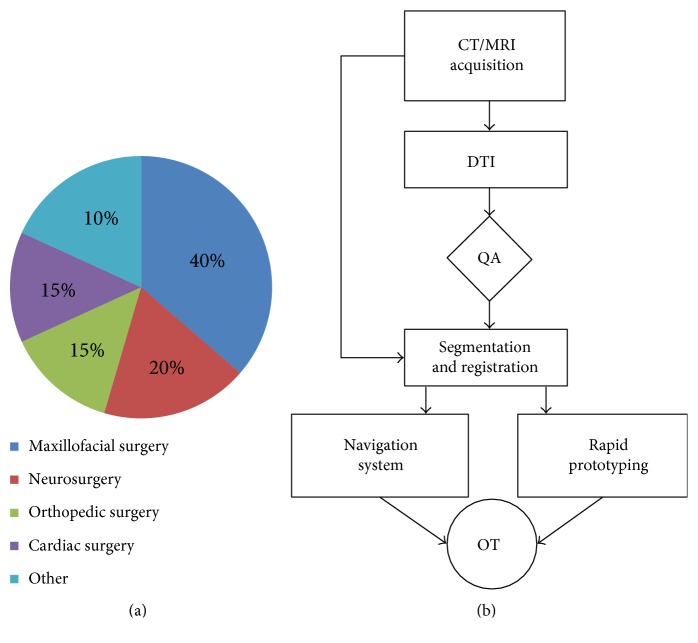
Clinical areas associated with the 200 surgeries assisted with 3D-printed models (a). Block diagrams showing the different steps required to create a 3D-printed model based on CT, MRI, and DTI data.

With the aims of improving surgical outcomes, reducing future costs, and developing thorough clinical guidelines for enhancing surgical planning and assessment, the National University Hospital of Iceland, Landspitali, established an in-house service for 3D rapid prototyping in 2007. Since its introduction, this service has allowed physicians and surgeons from different specialities to submit requests for a host of 3D models to be made available within 24 hours of submission. This process was simultaneously employed in research activities to study both the anthropometry of human muscles [11] and the use of rapid prototyping as preparation for complex brain surgeries in combination with neurosurgical navigation systems [12]. Since then, the National University Hospital of Iceland has fabricated over 200 surgical models for patient cases in the fields of cardiac, orthopedic, and neurosurgery (Figure 1(a)). The overwhelming success of this 3D rapid prototyping service has led to its solidification as an essential service within the hospital, and the rapid prototyping service continues to expand its impact on an increasing number of assisted surgical cases [13].

In neurosurgery, one technique that has likewise been increasingly used for preoperative planning is diffusion tensor imaging (DTI) tractography or fiber tracking. Tractography is a noninvasive technique that allows for the in vivo localization of fiber tracts in the brain. Tractography uses DTI, which is based on magnetic resonance imaging (MRI), to map brain connectivity, which can provide neurosurgeons with the opportunity to visualize nerve fiber tracts before surgery [14]. More specifically, this technique may be applied to that of functional MRI (fMRI), which has shown great utility in the context of surgical planning. fMRI utilizes hemodynamic responses within the brain to implicate regional recruitment with a variety of cortical functions, such as motor control and language processing [15]. Unfortunately, the routine integration of surgical planning, DTI, and preoperative fMRI has been primarily limited by concerns regarding acquisition and registration reliability. Nonetheless, there is much promise in this regard—evidenced most relevantly in the reconstruction of cotricospinal tracts for preoperative tumor planning [16, 17]. The purpose of this paper is to detail a novel approach to neurosurgical planning via the use of 3D printing, which combines patient-specific anatomy from traditional computer tomography (CT) and MRI images, with brain function derived from the in vivo localization of fiber tracts in the brain using DTI.

## 2. Material and Methods

The procedure of creating the 3D-printed models based on CT, MRI, and DTI data can be seen in Figure 1(b).

### 2.1. CT and MRI Acquisition

CT data were acquired from a Philips/Brilliance 64, the Head scan protocol was set to 119 mA X-ray for the tube current and 120 KV for tube voltage, and the slice thickness is typically between 0.6 and 1 mm.

MRI data were acquired from a 1.5T Siemens Avanto and the head coil used was Head Matrix Coil from Siemens. Both anatomical images and DTI were acquired for this process. The DTI protocol included a spin echo-echo planar imaging- (SE-EPI-) based DTI sequence with 20 diffusion directions, two repetitions to boost the SNR and *b* value (*b* is the diffusion sensitivity) equal to 1000 s/mm^2^. The anatomical image protocol included a T1-weighted 3D magnetization-prepared rapid acquisition gradient echo sequence (MP-RAGE).

### 2.2. DTI: Fiber Tract Extrapolation

Two different software (StealthViz [18] and nordicBrainEx [19]) programs were used to extrapolate the optimal fiber tracts for planning and rapid prototyping.

The fiber tracts of major interest for this process are the so called *eloquent fiber tracts*; these tracks are easily clinically assessed and are most important for the patient outcome. In total, five fiber tracts were extrapolated from both software platforms:

- Corpus callosum: the corpus callosum is located in the center of the brain and forms the largest white matter bundle. Its role is to transfer information between the left and right cerebral hemispheres [20].
- Motor tracts: they originate in the motor cortex area and descends down to the brain stem and spinal cord to control *α*-motor neurons. They can control posture, reflexes, and muscle tone as well as conscious voluntary movements [21].
- Sensory tracts: they are responsible for the sense of touch. They receive incoming messages for touch and limb movements from the body [22].
- Optic tracts: they transfer the information from the retina to the visual cortex of the brain [23, 24].
- Broca's area to Wernicke's area: arcuate Fasciculus is the prominent fiber tracts that connect these two areas that play a role in our language and speech [25].

#### 2.2.1. StealthViz

StealthViz is a surgical planning software application. It allows import of Digital Imaging and Communications in Medicine (DICOM) datasets that can be reviewed in 2D and with 3D volume rendering, multimodality image fusion, and segmentation of structures with manual and semiautomatic tools. The software performs white matter tractography. It enables realignment of diffusion-weighted gradient, coregistration with other anatomical and functional datasets, and tensor calculations. The fiber tracking uses deterministic FACT algorithm [26]. The workflow is the following:

- Import data: the MRI data are imported in DICOM format. Anatomical and diffusion tensor images are merged and the diffusion tensor positioned in the correct anatomical position.
- Segmentation: StealthViz allows segmentation with five different tools; pick region tool, brush tool, lasso tool, magic wand, and blow. A brain tumor can be segmented by using a *blow tool* which marks the region of interest on one cross section. The process can be iterated on several slices and those marked regions can be interpolated creating a 3D object of the tumor.
- Fiber tracking: to trace tracts in StealthViz a *start box* (and eventually a middle and end box) can placed on specific regions of interest in the brain, called seeding point, for example, in our application, we start in the region of corpus callosum. Then, the software computed all the fibers that go through the designed *box*. Different combinations of the boxes can be used to find the tracts of interest. Tracks that are not of interest can be removed. Calculated fiber tracts are visualized within the structural images both in 2D and in 3D.
- Calculate as 3D object: when the tractography planning is completed and approved by neurosurgeon, the tracts are converted in 3D objects and saved in a DICOM format. In this phase, an error margin of 1 mm is added to each fiber tract.
- Export planning: results can be exported as a one file or separated files (each for every track) to the surgical navigation system or to a USB flash memory.

#### 2.2.2. NordicBrainEx

NordicBrainEx is DICOM compatible and can analyze DTI data acquired with all major MRI scanners. DTI datasets acquired with two different *b* values (one *b* = 0 and six or more DWI where  *b* ≠ 0) can be analyzed in nordicBrainEx. The DTI analysis in nordicBrainEx generates parametric maps of various attributes of the diffusion tensor, including eigenvector color map (cDTI), fractional anisotropy index (FA), mean diffusivity (ADC), tensor eigenvalues (*λ*_1_, *λ*_2_, and *λ*_3_), and trace weighted (TraceW). The fiber tracking is performed by using FACT [26]. The workflow is the following:

- Import data: an automatic registration allows to place DTI data correctly according with the structural images.
- Fiber tracking: to perform tractography planning 5 different geometrical shapes can be selected for fiber tracking; ellipsoid, cube, polygon, free hand, or scatter. These geometrical shapes are used to define volume of interest (VOI) and find the tracts of interest. On a defined VOI, three logical operators are available: (1) AND which only visualize fibers passing through that VOI, (2) OR which will only visualize fibers passing through this and any other VOIs defined, or (3) NOT that will disregard all fibers passing through that VOI. When finished tracking, one fiber, for example, corpus callosum, can be saved individually.
- Export planning: results can be exported as separated files (each for every track) to the surgical navigation system or to a USB flash memory.

### 2.3. Quality Assessment: Anatomical Accuracy and Incorrectly Displayed Fibers

It is known that the different surgical planning software for fiber tracks may provide different results even though they are based on the same reconstruction algorithm [27]. For this reason, we performed a comparison between the software available in our institution to find the optimal one for rapid prototyping application. The assessment is based on anatomic accuracy and incorrectly displayed fibers for each fiber tract. These comparisons are done by grading the fiber-tracking results.  Table 1 shows the grading for incorrectly displayed fibers and Table 2 shows the grading for anatomic accuracy. The grades vary from 1 (best) up to 4 (worst). A white matter atlas was used as a reference for the evaluation [28, 29].

### 2.4. Segmentation and Registration

The next step is to combine the anatomical data such as the skull and other regions of interest with the tracks from the DTI software. We use for this propose the software MIMICS [30] that is a platform for medical image processing. The process can be divided in three steps:

*Step 1*. CT data are imported in MIMICS and the skull bone is segmented. This operation is threshold based; where a range of HU (typically, from 600 to 2000) values are selected that allow to display the bone tissue. Next we apply *region growing* to assemble the entire connected pixel within the defined threshold in a so called *mask*. Now a 3D object can be created directly from the mask and further modification (such as opening the skull model to see inside) can be applied on the 3D object using CAD tools. Finally the 3D model can be saved as standard tessellation language (STL) file which is a format compatible with 3D Printing technologies.

**Figure 2:**
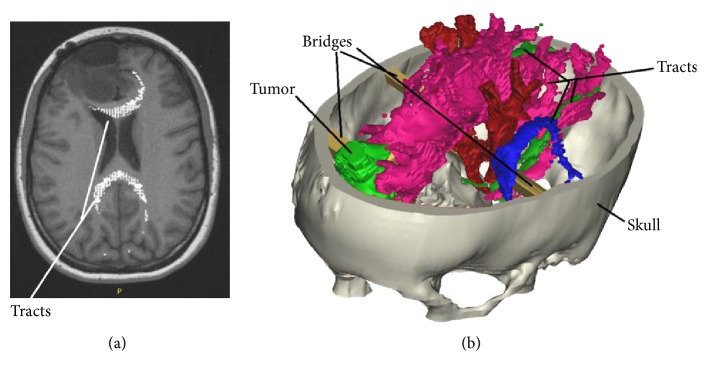
TRACTS superimposed on MRI structural data (a). 3D model including skull from CT, tumor from MRI, fiber tracts, and connecting bridges (b).

*Step 2*. The next step is to import the tractography DICOM files to MIMICS. The 5 tracks of interest are superimposed on the anatomy (MRI data) but appearing brighter compared to those of the surrounding tissue (Figure 2(a)); therefore, the threshold-based segmentation of each tracts is easy. The five 3D objects associated with each tracks were created in the same way as described in step 1. In order to improve the quality of the 3D objects for 3D printing, we applied some morphological operations on the mask in order to smoothen details equal or below to 0.25 mm and closing distance equal to 2.5 mm (holes or gaps of 0.25 mm or less are filled). Finally, the 3D model can be saved as STL file.

*Step 3*. The final step is to combine Tracts, MRI, and CT data within the same 3D object. First, we imported the MRI T1-weighted images to MIMICS. Soft tissues like tumor are better visualized with MRI, and therefore, the segmentation and creation of the 3D object for this region of interest is done in this phase using the same threshold-based procedure described above. Next, we import the STL files of the skull and fiber tracts. Fiber tracts were positioned in a semiautomatic way on the 2D structural images by projecting the contours from the 3D object of the tracts. Next, we imported the STL file of the skull. Since the CT and MRI data have different coordinate system, the skull 3D object is registered manually using a 3D-positioning panel. When the skull is in the right position, then the necessary connections (bridges drawn manually) between tumor, fiber tracts, and skull are built in order to create a 3D model that can be printed. Finally, the skull, tumor, fiber tracts, and the bridges are combined in one 3D object using *Boolean operations*, and the results are saved as STL (Figure 2(b)).

### 2.5. Navigation System and Rapid Prototyping

The 3D model of Figure 2 is finally printed using a ProJet® (3D systems, Rock Hill, USA) printer using a material called VisiJet®M3-X which is an organic colorless mixture that allow rendering of small details (mm scale). After print, the model is hardened with an infrared light.

The computer model can be exported to the surgical navigation system as DICOM set, StealthStation® [31], which works as a GPS system determining the position of surgical instruments in relation to patient images by automatically fusing CT and MRI scans. Then, a registration is done with patient anatomy; so, there is a linkage between the patient and the system. In this application, we use the 3D-printed model instead of the real patient; in this way advanced preparation of the surgery can start before the patient enters the operation theatre.

## 3. Results

We validate this process collaborating to a neurosurgical planning of a 29-year-old female having a low-grade glioma located on the frontal lobe.

**Figure 3:**
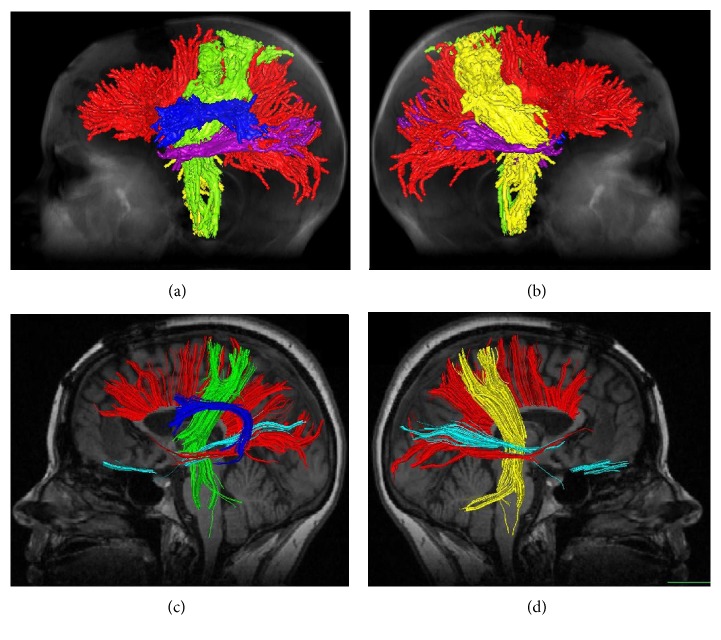
Tractography planning from StealthViz (a-b) and nordicBrainEx (c-d). Red color represents fiber tracts from corpus callosum, green color the tracts from motor and sensory area on the left side, and yellow the same tracts on the right side. Dark blue represents the arcuate fasciculus and purple shows the optic nerves.

**Figure 4:**
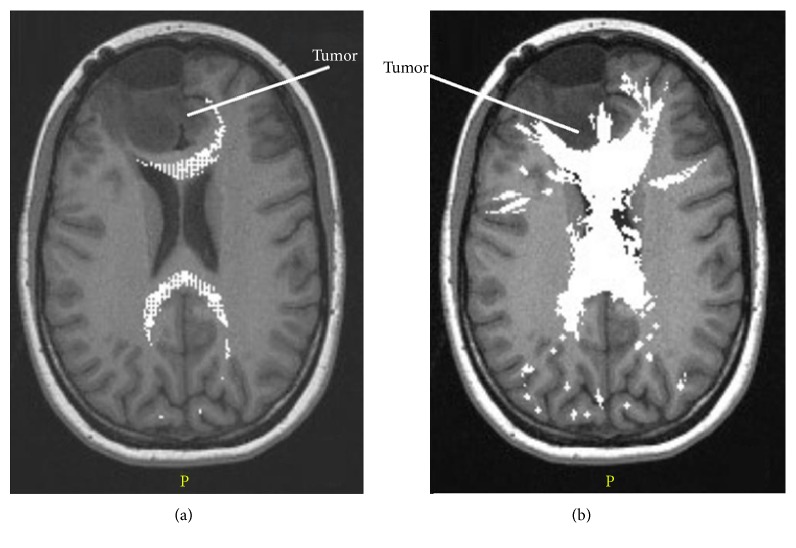
Comparison between the corpus callosum tracts obtained with different software: nordicBrainEx (a) and StealthViz (b). Both (a) and (b) show the same slice.

**Figure 5:**
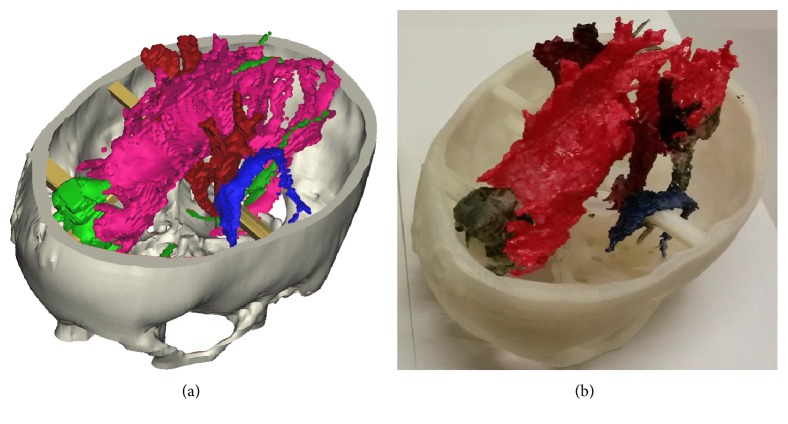
3D computer model made in mimics with fiber tracts result from nordicBrainEx (a) and when it has been 3D printed and painted (b).

The five fiber tracts that we focused on in this study can be seen in Figure 3, it shows the side views tractography planning from the two surgical planning software platforms: Figures 3(a) and 3(b) for StealthViz and Figures 3(c) and 3(d) for nordicBrainEx. It can be noticed that the pathways for the tracts are similar but not the identical; indeed, there are remarkable differences in thickness and ending morphology between the software platforms that may be important in relation to the pathological area of interest. In order to assess the results from the two fiber tracts planning, we use image comparison software called XERO viewer [32]; here, the fiber tracts, superimposed on the MRI data, were viewed simultaneously and visually assessed.  Figure 4 shows the comparison of corpus callosum. To be noticed, the surface of corpus callosum is shown in Figure 4(b) that displays a false positive. Moreover, the fiber tract from StealthViz goes out of white matter in the brain and it is difficult to assess the exact position. Based on comparison, slice by slice and tract by tract, we assess the two tractography planning based on anatomic accuracy and incorrectly displayed fibers [28, 29]. The quantitative results are displayed in Table 3 where for this study case, the tractography plane made with nordicBrainEx has a better score and was chosen for the next step.  Figure 5 shows the computer model (a) and 3D-printed model (b) resulting from the nordicBrainEx surgical planning.

DTI planning and 3D-printed models were used with the neurosurgical navigation system [12] to prepare the surgical operation where the tumor was removed from the frontal lobe. The operation was successful, and advanced planning provided with DTI planning and 3D models allowed the neurosurgeons to be better prepared during surgery.

## 4. Conclusion

Three-dimensional models and navigation systems for neurosurgery can be combined to improve surgical planning and surgeon training [12, 32]. The work reported herein demonstrates that preoperative planning using diffusion tensor imaging (DTI) tractography and 3D models is feasible and can be employed in the preparation of complex operations. Additionally, it is likely that this process can shorten operation times, contribute to better patient safety, and be used for training surgeons.

Even though DTI tractography is not a fully reliable method, it can still provide the neurosurgeons with an overview of fiber tract position, and it has been shown that the use of DTI improves tumor resection results and decreases postoperative deficits [14, 33]. Altogether, this work demonstrates that the reported 3D-printing process may be integrated with DTI planning and add valuable information for neurosurgical planning—especially in association with surgical navigation systems.

## Figures and Tables

**Table 1:** Grades for incorrectly displayed fibers.

Incorrectly displayed fibers	
Grade 1	None
Grade 2	<10% of all displayed fibers
Grade 3	<25% of all displayed fibers
Grade 4	>50% of all displayed fibers

**Table 2:** Grades for anatomic accuracy.

Anatomic accuracy	
Grade 1	Follow fiber tracts within anatomical boundaries
Grade 2	Follow fiber tracts outside anatomical boundaries
Grade 3	Follow poorly anatomical fiber tracts
Grade 4	Do not follow anatomical fiber tracts

**Table 3:** The grading results for anatomic accuracy and for incorrectly displayed fibers for both StealthViz (SV) and nordicBrainEx (BE).

Nerve tracts	Anatomic accuracy		Incorrectly displayed fibers	
	BE	SV	BE	SV
Arcuate fasciculus	3.5	1.0	3.5	3.0
Corpus callosum	2.0	1.0	2.0	1.5
Left motor and sensory tracts	2.0	1.5	2.0	1.5
Right motor and sensory tracts	2.0	1.5	2.0	1.5
Optic tracts	4.0	3.0	4.0	4.0
Total	13.5	8	13.5	12.5
